# Assessing electronic device use behaviours in healthy adults: development and evaluation of a novel tool

**DOI:** 10.1186/s12889-024-17637-4

**Published:** 2024-01-15

**Authors:** Naomi K. Fitzpatrick, Veronique Chachay, Sandra Capra, David Briskey, Sarah Jackman, Angela Shore, Joanna Bowtell

**Affiliations:** 1 Sport and Health Sciences, University of Exeter, Exeter, UK; 2 School of Human Movement and Nutrition Sciences, The University of Queensland, St Lucia, QLD Australia; 3 NIHR Exeter Clinical Research Facility, University of Exeter, Exeter, UK

**Keywords:** Blue light, Screen time, Survey methods, Questionnaire designs, Macular degeneration

## Abstract

**Background:**

Chronic exposure of the macula to blue light from electronic devices has been identified as a potential macular health concern. The impacts remain poorly investigated as no validated methods to capture usual device use behaviours exist.

**Purpose:**

The aim of this study was to develop and validate the Electronic Device Use Questionnaire (EDUQ) against multiple 24-h electronic device use diaries in healthy Australian and United Kingdom adults.

**Methods:**

The EDUQ and diaries were developed to capture device use across categories (television, computer and handheld devices). Over eight weeks 56 Australian and 24 United Kingdom participants completed three questionnaires and eight diaries via online platforms. Tool validity was determined through Bland–Altman plot analysis of mean daily hours of device use between the tools.

**Results:**

The EDUQ demonstrated poor validity in both cohorts with poor agreement when compared with the diaries. When the device categories were combined, a mean difference between the tools of 1.54 h/day, and 95% limits of agreement between -2.72 h/day and 5.80 h/day was observed in the Australian cohort. Across both cohorts and all device categories the mean differences indicated individuals were more likely to report higher device use through the questionnaire rather than diaries.

**Conclusions:**

The EDUQ is a novel tool and demonstrated the difficulty for participants of accurately recalling usual behaviour of device use. Poor agreement in reported device use occurred across all device categories. The poor agreement may be related to factors such as memory recall bias, and the number of diaries captured not being reflective of usual use. Future studies should look to address these factors to improve validity of device use capture.

**Supplementary Information:**

The online version contains supplementary material available at 10.1186/s12889-024-17637-4.

## Background

Prolonged and chronic exposure to electronic devices, referred to as ‘devices’ hereinafter, has been identified as an emerging public health issue with implications for conditions such as sleep issues, digital eye strain (also known as computer vision syndrome), myopia, and retinal damage in the eye [1–4]. The exposure to blue light from device screens has been hypothesised to cause photochemical damage at the macula in the eye [5, 6]. Chronic exposure to blue light from devices has not yet been confirmed as a radiation issue; however, investigation is warranted due to the plausible mechanism for retinal damage supported by animal studies. Photochemical damage to the retina from blue light has been demonstrated in both in vitro and animal experimental studies [7–9]. Additionally, the light emitting diode form of blue light exposure seen from devices is a relatively new environmental exposure with no longitudinal data available on the potential impacts.

Devices in this study refer to those with display screens such as smartphones, tablets, computers, and televisions. The impact of long-term human blue light device exposure has not yet been investigated, in part because no validated methods to measure this human exposure exist. Reports to date have been with unvalidated interview or questionnaire methods, and often through commercial entities. The 2019 Deloitte mobile and media report is one such example and indicates that the uptake and use of devices has increased since 2017. The report indicated that nine in 10 Australians own a smartphone, and average daily use is three hours [10, 11]. The Deloitte Media and Entertainment Consumer Insights 2023 report indicated that Australian adults spend 3 h and 54 min per day watching videos, 54 min per day browsing social media, and 30 min per day playing video games [12]. Another commercial report, the United Kingdom (UK) based Ofcom 2018 Communications Market Report, indicated from self-reported recall that one in five adults spent a weekly average time online (activities involving internet use) of more than 40 h [13].

The use of devices appears to be widespread; however, behaviours surrounding the types of devices being used and habitual patterns of use are unclear. A specific and valid method for monitoring device use behaviours is needed to understand behaviour patterns. A method is also needed to determine the clinical implications of the potential negative impacts of blue light exposure, such as myopia and macular degeneration risk [1–3]. In addition to ocular health implications, a method to monitor device use behaviours may have application in other areas of research such as use of devices as assistive technology, social equity, and psychosocial impacts on interpersonal relationships [14–16]. This study describes the development and validity evaluation of a novel tool to monitor usual device use titled the Electronic Device Use Questionnaire (EDUQ). The study aims were to develop the EDUQ and validate daily hours of device use reported by the EDUQ against multiple 24-h electronic device use diaries (24DUD) in healthy Australian and UK adults.

## Methods

### Recruitment

A convenience sample of adults residing in Australia and the UK was recruited via electronic and paper advertisements. Australian participants were recruited between August 2020 and June 2021, and UK participants were recruited between August 2021 and November 2021. Eligible participants were healthy adults 18 years or older able to complete online questionnaires. The exclusion criteria were no English language literacy and visual, hearing, or physical impairment that prevented online questionnaire completion. This study was approved by the University of Queensland Low and Negligible Risk ethics committee and the Sport and Health Sciences ethics committee at the University of Exeter (#2020001764). All participants provided written informed consent.

### Electronic device use questionnaire development

As no literature specifically addresses valid capture of screen time from devices, the literature on research in physical activity, dietary intake, and myopia was drawn upon [3, 17, 18]. Five key factors for consideration in the development of the questionnaire emerged from this literature: the categories of devices, day-to-day variability in device use, timeframe of participant recall, question structures to report device use, and other daily behaviours that may inform device use [3, 17, 18].

The categories of devices aimed to capture differences between devices in patterns of use, device screen luminance, and distance of viewing from device [19]. The luminance of a device and viewing distance from a device during use may play a role in their impact on ocular health, for example smartphones may have a lower luminance compared to a television but are held a shorter distance from the eye [19]. Thus, three logical categories were handheld devices (for example, smartphones and tablets), computers (for example, laptops and desktop monitors), and televisions (including household and commercial sizes). This grouping was adopted from the device groupings in the three device use related questions in the University of Houston Near work Environment Activity, and Refraction (UH NEAR) questionnaire. The UH NEAR was developed to investigate near viewing activities such as reading, writing, and use of devices [3].

Day-to-day variability in device use is a likely bias equivalent to that established in other areas of behaviour research, such as dietary intake [13, 20, 21]. As with dietary intake, the day-to-day variability may be impacted by participant characteristics such as age and occupational status [13]. The need to capture day-to-day variability is also supported by prior research, where it has previously been estimated using the UH NEAR questionnaire that device use is approximately three hours more on a weekday compared to a weekend day [3].

The timeframe of participant recall was selected with consideration for the unknown degree of variability in device use behaviours, potential for episodic device use, and memory recall bias. The established biases and recall timeframe used in dietary intake and sedentary behaviour research informed the timeframe of recall for the EDUQ [18, 22]. A moderate length recall timeframe of 3 months was selected to balance the attempt to capture habitual device use whilst reducing the impacts of episodic behaviours, mathematical cognitive and calculation difficulty, and memory recall bias [17, 23, 24].

Question structure was considered so that use of devices over a day was captured [3, 18]. A parameter of 30-min intervals for reporting daily hours of use for each device was selected. A pre-determined range was selected to assist reducing the cognitive difficulty of recalling the behaviour [3, 18]. The UH NEAR questionnaire utilised 60-min intervals and the questionnaire returned high rates of overreporting compared to glasses that recorded distance of the eye from an object over the same recall period [3]. Activity diaries utilised 15- or 30-min intervals [18]. While fifteen-minute intervals may be appropriate for reporting episodical use of devices such as smartphones, however 15-min intervals may also require higher mathematical computational and averaging capacity, which may negatively impact the accuracy of recall [18]. Thus, a 30-min interval was selected for reporting hours of device use.

The final factor considered was other daily behaviours that may inform device use. As a novel area of behaviour research, other daily behaviours, and participant characteristics may be important to understand device use patterns. Auxiliary daily items included were physical activity, sleep, occupational status, duties within occupational role, history of device use, use of blue-light filtering ocular lenses and device settings, and device-generated reports of daily use [3, 25, 26].

An internal test of face validity was conducted with two members of the research team (S.C. and V.C.) and a convenience sample of 21 Australian and UK individuals known to N.F. who volunteered to read, fill out and discuss the EDUQ [27]. Discussions with respondents indicated all individuals understood what an electronic device is and that daily hours of device use were requested for a weekday and weekend day separately. All but two individuals reported the 30-min increment for reporting device use to be appropriate, while two respondents suggested a 15-min increment could improve the EDUQ. Three changes were made to the EDUQ following respondent feedback. One change was clarifying what constitutes physical activity through providing examples of activities. Another addition was including the daily hours of use as reported by the devices’ own data capture system (e.g. on a smartphone). The last change was providing examples of lutein and zeaxanthin containing supplements to assist recall of supplement intake. The final EDUQ contained four sections with a total of 22 questions (see Additional file 1). Section one contained nine questions relating to personal characteristics and medical history, including age, gender, country of residence, and ocular health. Section two contained three questions relating to education and occupational status. Section three contained five items relating to device use. Three categories of devices with screens were included: handheld devices (for example, smartphones and tablets), computers (for example, laptops and desktop monitors), and televisions (including household and commercial sizes). The items included reporting usual daily hours of device use on a weekday and a weekend day, change in daily device use over the last one to 20 years, and use of visual correction glasses with or without a blue light filter. Section four contained four questions relating to the use of sunglasses, physical activity and sleep on weekdays and weekend days.

### Twenty-four-hour electronic device use diary development

The 24DUD was developed to perform relative validity testing with the EDUQ, as no other tools designed specifically to monitor device use existed. The diary was developed by adaptation of a prospective physical activity diary used by Cartmel et al. [18]. This diary was modified to reflect electronic device use. Titled the ‘24-h electronic device use diary’, the diary recall timeframe was prospective from 00:00 to 23:59 and contained 15-min intervals in which participants recorded use of handheld, computer, and television devices (see Additional file 2).

### Data collection

**Fig. 1:**
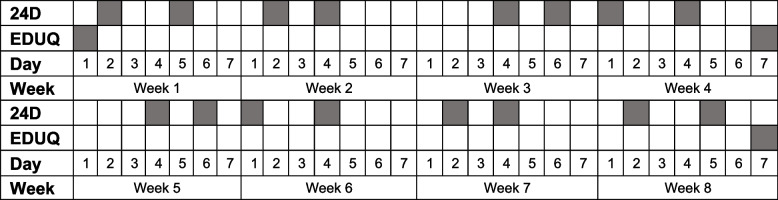
Questionnaire and diary schedule of data collection. The day of the week for the measurement of 24-h electronic device use diaries varied randomly between participants. Abbreviations: 24D, 24-h electronic device use diary; EDUQ, Electronic Device Use Questionnaire

Over eight weeks, recruited participants completed eight (one per week) diaries and three EDUQs (Fig. 1). The day for diary completion was randomly allocated at baseline within the constraints that two of the eight diaries were scheduled for weekend days and the remainder for weekdays. The EDUQ was completed at baseline and at the conclusion of weeks four and eight. Participants were notified by email when a diary or EDUQ was to be completed. The EDUQ and diary were hosted on Checkbox Survey® for Australian participants and Qualtrics XM® survey platform for UK participants.

### Data processing

In the EDUQ, mean daily hours of device use for each device category cumulatively and separately was derived using:

$$\mathrm{EDUQ\, mean\, daily\, hours }= ((\mathrm{Weekday\, device\, use }\times 5) + (\mathrm{Weekend\, day\, device\, use}\times 2))\div 7$$

(see Additional file 3). In the diaries, the mean daily hours of device use for each device category cumulatively and separately were derived using

$$\mathrm{Diary\, mean\, daily\, hours }=\mathrm{ Sum\, hours\, from\, all\, completed\, diaries }\div \mathrm{ number\, of\, diaries\, completed}$$

### Sample size

In the absence of a validated tool or literature on device use, physical activity and near viewing activity questionnaire literature was referenced to determine a sample size. One study demonstrated that 24 adults aged 66–88 years was a sample size able to indicate reporting trends between two tools with the comparison of a physical activity questionnaire to an activity diary [18]. The validation study of the UH NEAR questionnaire by Williams et al. [3] had a sample size of 23 participants and was able to obtain an indication of questionnaire validity but suggested that a larger sample size would be beneficial for future studies. Thus, a minimum goal sample size of 40 participants per country (Australia and UK) was determined.

### Statistical analyses

Statistical analysis was conducted using SPSS (28.0.0.0) [28]. Participant responses to each EDUQ were screened for likely overreporting by summing the responses to daily hours of device use, physical activity, and sleep. A sum over 168 h/week was flagged and investigated further, as participants could have overreported one or all three behaviours. Other participant characteristics, such as occupation, were reviewed to determine the feasibility of high device use contributing to the more than 168 h/week. Participants with 172 or less hours/week and plausible characteristics to explain high device use were included in the questionnaire analysis. Any participant with EDUQ reporting over 168 h per week and no feasible explanation was excluded. The 24DUDs were assumed to be accurate and included as long as the participant reported one or more EDUQ that passed the screening process for overreporting.

Data normality was tested with the Shapiro–Wilk test. Differences between cohort participant characteristics and device use were tested with a Chi-squared test, two-tailed independent samples t–test or Mann–Whitney U–test. In both cohorts, a Bland–Altman plot analysis of the mean daily hours of device use (all categories combined) was performed to compare the third EDUQ and six or more combined 24DUDs [29, 30]. The third EDUQ was used so that the timeframe of recall for EDUQ device use aligned with reporting from the diaries. The same Bland–Altman plot analysis was also performed for each device category individually. Participants with fewer than six 24DUDs were removed from the questionnaire analysis. Six rather than eight 24DUDs were chosen to increase the data available for analysis, as only seven UK participants had completed all eight diaries. Six diaries were determined to be appropriate, as no significant difference was found between the complete or partially complete larger Australian dataset for the parameters required for the Bland–Altman plot analysis. If the difference between tools was not normally distributed, the data were log base 10 transformed to achieve normality for Bland–Altman plot analysis. Cronbach’s alpha and two-way mixed effects model absolute intraclass correlation coefficient was performed for test–retest reliability between the first, second, and third EDUQ. Normally distributed data are presented as the mean ± standard deviation, and nonnormally distributed data are presented as the median and 25th to 75th percentile. The results were considered statistically significant at *p* < 0.05.

## Results

**Fig. 2:**
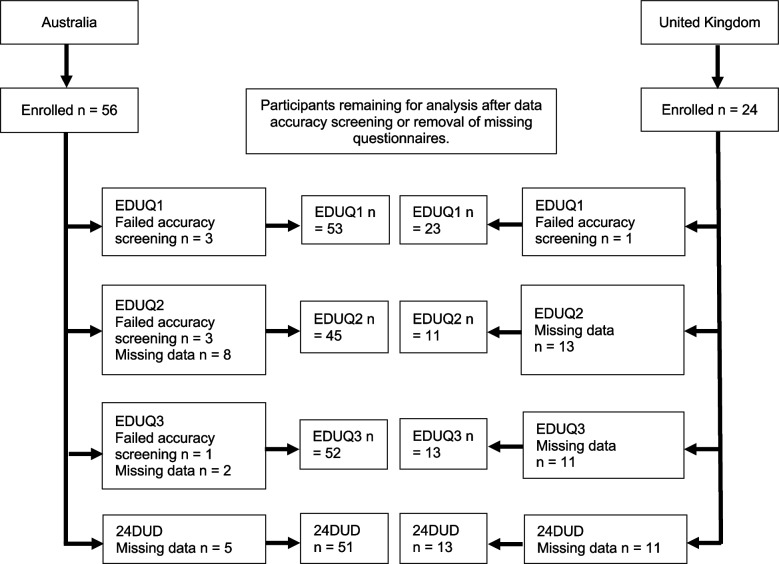
Participant flow chart of device use study completion. Abbreviations: n, number of participants; EDUQ, Electronic Device Use Questionnaire; 24DUD, 24-h electronic device use diary

Fifty-six Australian and 24 UK participants enrolled in the study. Across the third EDUQ and diaries, six Australian and 11 UK participants had implausible EDUQ data or did not complete the questionnaires needed for the validity and reliability analysis (Fig. 2).

The median age of the Australian participants was 27 (25 – 32) years, 68% were female, and 88% had a tertiary education (Table 1). The median age of the UK participants was 27 (25 – 52) years, 63% were female, and 54% had a tertiary education. Significant differences in age (*p* = 0.002), body mass index (*p* = 0.02), and education status (*p* < 0.001) were present between the Australian and UK cohorts.

**Table 1:** Australian and United Kingdom participant characteristics

	Median (25th – 75th percentile)		Difference between cohorts
	Australian (*n* = 56)	UK (n = 24)	
Age (years)	27 (25 – 32)	27 (25 – 52)	*p* = 0.002
Sex (% female)	68%	63%	*p* = 0.29
BMI (kg/m^2^)	24 (22 – 26)	26 (24 – 31)	*p* = 0.02
Physical activity per week (hours)	5 (3 – 8)	3 (0.5 – 7)	*p* = 0.06
Sleep per night (hours) (mean ± SD)	7.7 ± 0.73	7.0 ± 0.97	*p* = 0.002
Education (% completed higher education)	88%	54%	p < 0.001
Occupational status (% student, % employed)	49%, 46%	25%, 58%	p = 0.07

Difference between cohorts tested by Mann–Whitney U-test for continuous variables and Chi-squared test for categorical variables. *Abbreviations n* number of participants, *UK* United Kingdom, *BMI* Body mass index, *SD* Standard deviation

The mean Australian device use reported from the EDUQ ranged from 8.9 to 9.6 h/day. The mean UK use ranged from 11.1 to 11.7 h/day (Table 2). Computers were the device category with the highest mean daily use across both cohorts and tools. Australian reported hours of use for all device categories individually and combined were significantly correlated between the third EDUQ and 24DUDs (Table 3). Of both cohorts, the strongest correlation was in the UK cohort with handheld device use, r = 0.93, R^2^ = 0.87 (*p* < 0.001).

**Table 2:** Daily hours of electronic device use reported from the Electronic Device Use Questionnaire and mean of combined 24-h electronic device use diaries in the Australian and United Kingdom cohorts

Tool	Device category	Australia		United Kingdom		Cohort comparison ^a^
		*n* =	Daily Use (hours)	*n* =	Daily Use (hours)	
EDUQ 1	All devices	53	8.9 ± 3.16	23	11.4 ± 3.25 ^b^	*p* = 0.002
	Television		1.1 (0.50 – 2.75)		2.4 (1.50 – 4.00) ^c^	*p* = 0.008
	Computer		5.1 (3.40 – 6.60) ^d^		4.6 ± 2.98	
	Handheld		2.3 (1.29 – 3.18)		3.2 (2.00 – 6.64)	*p* = 0.048
EDUQ 2	All devices	45	9.2 ± 3.08 ^e^	11	11.7 ± 2.60 ^f^	*p* = 0.01
	Television		1.5 (0.61 – 2.57)		2.0 ± 1.51	
	Computer		4.7 ± 2.17		5.8 ± 2.64	
	Handheld		2.8 ± 1.65		3.8 ± 3.05	
EDUQ 3	All devices	53	9.6 ± 2.61 ^g^	13	11.1 ± 2.22	*p* = 0.04
	TV		1.5 (0.50 – 2.57)		2.5 ± 2.11	
	Computer		4.9 ± 1.76 ^h^		4.8 ± 3.42	
	Handheld		3.0 (1.68 – 3.79)		3.9 ± 3.12	
Mean 24DUD	All devices	51	7.9 ± 1.75 ^e, g^	13	9.3 ± 2.21 ^b, f^	
	TV		1.5 (0.90 – 2.38)		1.6 ± 1.55 ^c^	
	Computer		4.0 ± 1.78 ^d, h^		4.0 ± 3.46	
	Handheld		2.3 ± 1.31		3.6 ± 3.4	

Data presented as mean ± SD or median (25th – 75th percentile). Differences between countries tested by two-tailed independent samples t-test or Mann–Whitney U-test. Within country differences between questionnaires for a device category tested by two-tailed independent samples t-test or Mann–Whitney U-test and indicated by matching superscript letter (for example, ^b^)

*Abbreviations:* *EDUQ* Electronic Device Use Questionnaire, *24DUD* 24-h electronic device use diary, *n* Number of participants

^a^ Blank cell indicates non-significant differences between cohorts for row variable. ^b^ *p* = 0.049. ^c^ *p* = 0.047. ^d^ *p* = 0.02. ^e^ *p* = 0.02. ^f^ *p* = 0.04. ^g^ *p* < 0.001. ^h^ *p* = 0.007.

**Table 3:** Bland–Altman plot analysis outcomes of daily hours of electronic device use reported from the Electronic Device Use Questionnaire and 24-h electronic device use diaries

		Device category	Bland–Altman Plot Analysis (hours / day)			Correlation between reported use
			Mean difference (95% CI)	Lower 95% LOA (95% CI)	Higher 95% LOA (95% CI)	
Australia	EDUQ3 vs 24DUD (*n* = 50)	All devices	1.54 (0.00 – 3.08)	-2.72 (-4.26 – -1.18)	5.80 (4.26 – 7.34)	r = 0.54, R^2^ = 0.29 *p* < 0.001
		Television ^a^	0.08 (0.00 – 0.16)	-1.59 (-1.67 – -1.51)	1.74 (1.67 – 1.82)	r = 0.79, R^2^ = 0.64, *p* < 0.001 ^b^
		Computer	0.95 (0.00 – 1.90)	-2.28 (-3.23 – -1.33)	4.18 (3.23 – 5.13)	r = 0.57, R^2^ = 0.33, *p* < 0.001
		Handheld ^c^	0.14 (0.00 – 0.30)	-0.32 (-0.40 – -0.22)	0.91 (0.67 – 1.18)	r = 0.80, R^2^ = 0.64, *p* < 0.001 ^b^
UK	EDUQ3 vs 24DUD (*n* = 12)	All devices	1.98 (0.00 – 3.97)	-2.80 (-4.78 – -0.87)	6.77 (4.78 – 8.75)	r = 0.44, R^2^ = 0.19, *p* = 0.16
		Television	0.72 (0.00 – 1.45)	-1.54 (-2.26 – -0.82)	3.76 (3.03 – 4.48)	r = 0.57, R^2^ = 0.33, *p* = 0.05
		Computer ^c^	0.15 (0.00 – 0.32)	-0.77 (-0.80 – -0.73)	4.67 (3.93 – 5.52)	r = 0.84, R^2^ = 0.71 *p* = 0.001
		Handheld ^c^	0.26 (0.00 – 0.60)	-0.36 (-0.49 – -0.19)	1.49 (0.97 – 2.14)	r = 0.93, R^2^ = 0.87, *p* < 0.001

Australian cohort EDUQ3 and 24DUD all devices: SEM = 0.31, t value (49 df) = 5.01. Australian cohort EDUQ3 and 24DUD TV: SEM = 0.12, t value (49 df) = 0.65. Australian cohort EDUQ3 and 24DUD Computer: SEM = 0.23, t value (49 df) = 4.08. Australian cohort EDUQ3 and 24DUD Handheld: SEM = 0.02, t value (49 df) = 3.57. UK cohort EDUQ3 and 24DUD all devices, SEM = 0.70, t value (11 df) = 2.81. UK cohort EDUQ3 and 24DUD TV, SEM = 0.73, t value (11 df) = 2.17. UK cohort EDUQ3 and 24DUD Computer, SEM = 0.10, t value (11 df) = 0.60. UK cohort EDUQ3 and 24DUD Handheld, SEM = 0.04, t value (11 df) = 2.34

*Abbreviations: CI* Confidence interval, *EDUQ* Electronic Device Use Questionnaire, *n* Number of participants, *24DUD* 24-h electronic device use diary; LOA, limit of agreement; SEM, standard error of the mean; df, degrees freedom; UK, United Kingdom

^a^ Indicates the analysis was performed with a difference that was not normally distributed and data transformation did not improve. ^b^ Indicates Spearman’s rank correlation test rather than Pearson. ^c^ Log base 10 transformation of data required for difference between tools to be normally distributed, values reported are back transformed

**Fig. 3:**
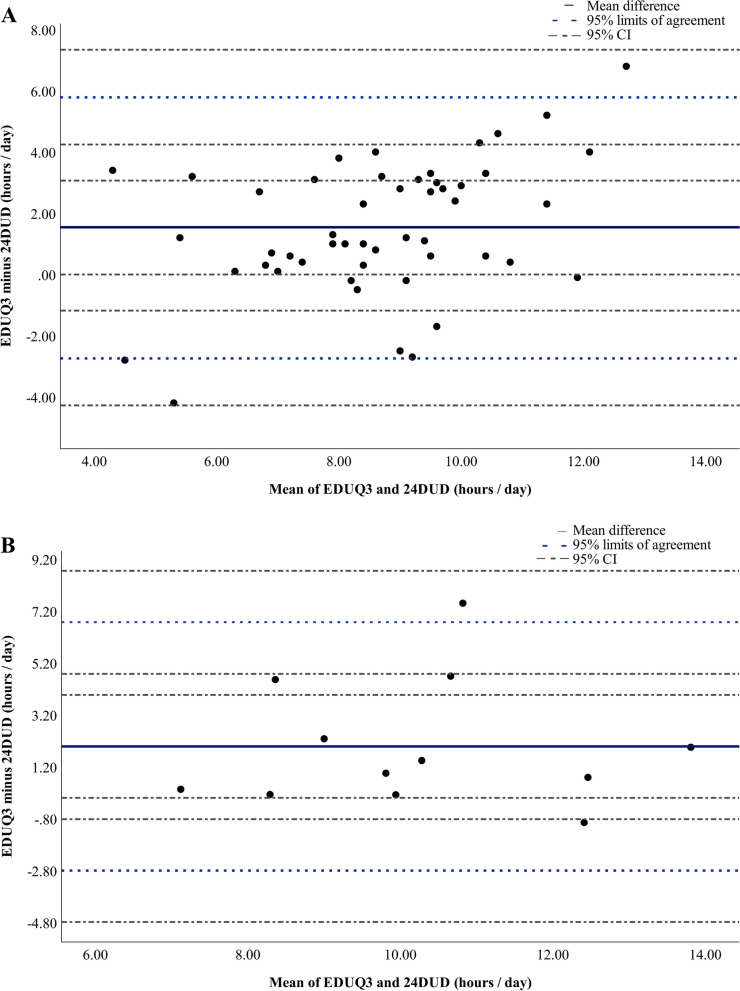
Bland–Altman plot analysis, EDUQ3 all devices combined compared with 6 or more 24-h diaries. **A**, Australian cohort. **B**, United Kingdom cohort. Abbreviations: EDUQ3, third Electronic Device Use Questionnaire; 24DUD, 24-h electronic device use diaries

For both cohorts, the Bland–Altman plot analysis indicated poor agreement of daily hours of ED use between the third EDUQ and combined 24DUDs with modest mean differences but large 95% limits of agreement (Table 3). The Australian cohort indicated slightly better agreement than the UK cohort, with a mean difference of 1.54 h and 95% limits of agreement from -2.72 h to 5.80 h. There were no trends in the direction of differences between tools (Fig. 3).

The three EDUQs in the Australian and UK cohorts indicated moderate to high test–retest reliability. In the Australian cohort, the highest test–retest reliability was between the second and third EDUQ, with a Cronbach’s α = 0.91 and a two-way mixed effects model absolute intraclass correlation coefficient of 0.91. In the UK cohort, the equal highest test–retest reliability was between the first and third EDUQ and the second and third EDUQ, both with a Cronbach’s α = 0.92 and a two-way mixed effects model absolute intraclass correlation coefficient of 0.92 (Table 4). Despite these results, the EDUQ had a poor ability to rank participants into tertiles by daily hours of device use. In the Australian cohort, there was 25% to 36% misclassification of participants into adjacent or opposite tertiles when comparing the first, second, and third EDUQs. Additionally, when ranked by tertiles determined by the diaries, there was 50% misclassification of participants with their third EDUQ response.

**Table 4:** Test–retest reliability of the three Electronic Device Use Questionnaires completed with all device categories combined

		*n* =	Cronbach’s α	Absolute ICC (95% CI)	*p* value
Australia	EDUQ1 vs EDUQ2	44	0.78	0.78 (0.60 – 0.88)	< 0.001
	EDUQ1 vs EDUQ3	50	0.78	0.78 (0.61 – 0.87)	< 0.001
	EDUQ2 vs EDUQ3	44	0.91	0.91 (0.84 – 0.95)	< 0.001
UK	EDUQ1 vs EDUQ2	11	0.79	0.80 (0.25 – 0.95)	0.01
	EDUQ1 vs EDUQ3	13	0.92	0.92 (0.74 – 0.98)	< 0.001
	EDUQ2 vs EDUQ3	11	0.92	0.92 (0.71 – 0.98)	< 0.001

*Abbreviations UK* United Kingdom, *EDUQ* Electronic Device Use Questionnaire, *n* = number of participants, *ICC* Intraclass correlation coefficient, *CI* Confidence interval

## Discussion

The novel EDUQ was developed and evaluated against multiple 24DUDs in adults located in Australia and the UK. Predetermined limits of agreement did not exist on which to benchmark the validity of the EDUQ. Validity was therefore determined by whether the EDUQ agreement with the diaries was such that the EDUQ would be able to capture differences in device use in an intervention or observational study. The poor agreement observed between the third EDUQ and diaries indicated that the EDUQ is not yet valid for use (Table 3). In the Australian cohort, the mean difference (95% limits of agreement) was 1.54 h/day (-2.72 h/day to 5.80 h/day). The range between the limits of agreement was 8.5 h, which is nearly equivalent to the mean daily device use of 7.9 – 9.6 h/day measured from the two tools in this cohort (Table 2). The moderate to high test–retest reliability suggests that the EDUQ is reliable. However, the EDUQ had a poor ability to rank participants by daily hours of device use into tertiles between the first, second, and third EDUQ, confirming its inadequate validity. The differences in reported combined device use between the third EDUQ and diaries appear to be related to an accumulation of participant misestimation within each device category. Additionally, there appears to be no clear trends or predictability in the direction of reported differences across the spectrum of daily device use.

This is the first study to the author group’s knowledge that has developed and reported total daily hours of device use. As such, there is no existing peer-reviewed research available on total daily hours of device use to compare against. This study can be compared with prior commercial reports that use unvalidated interview and questionnaire methods. The 2019 Deloitte mobile and media reports indicated that the average smartphone use for Australians is 3 h/day, and the average television use is just over 3 h/day [10, 11]. In this study, the daily hours of handheld use were similar or lower, and television use was lower than that of the commercial report. The median daily handheld device use reported by the third EDUQ was the same at 3.0 h, and the mean from the 24DUDs was 0.7 h less. The median daily television use reported by the third EDUQ and 24DUDs were both approximately 1.5 h less. The UK-based Ofcom 2018 Communications Market Report indicated that one in five adults spend more than 40 h/week online on the internet, including all devices [13]. In the first EDUQ, the UK cohort indicated a mean of 79.8 h of device use per week, approximately 40 h more per week. However, this is inclusive of both online and offline activity. Therefore, the discrepancy may in part be explained by differences in the hours of online and offline device use. The discrepancy and outcomes of this study indicate that offline use may constitute a significant portion of daily device use. The discrepancy may also be explained by usual daily hours of device use continuing to increase. Compared to 1 year ago 32% of Australian and 50% of UK participants indicated an increase in ED use. In contrast, compared to 5 years ago, 72% of Australian and 88% of UK participants indicated an increased in their ED use (Additional file 4). Reasons for change in device use reported by participants included change to work or study requirements, increased accessibility to devices, increased functionality of devices (e.g. online newspapers), and more engagement with social media.

One reason for the poor agreement between the EDUQ and diaries may be the difference in intervals provided for participants to report their device use between the EDUQ and diaries. Participants could report hours of device use in 30-min intervals in the EDUQ and 15-min intervals in the diaries. The larger intervals in the EDUQ may have contributed to the higher mean daily hours of device use reported by the EDUQ compared to the diaries. Future studies should consider closer alignment reporting intervals between tools, for example, reporting intervals of 15 min for both the EDUQ and diary.

Another reason for the poor agreement is likely the memory recall bias of recalling device use retrospectively with the EDUQ. Memory recall bias is well established in other areas of behaviour research, such as dietary intake [17, 23]. The presence of memory recall bias with recalling device use is also supported by prior research investigating daily hours of ‘near and intermediate activity’ with the UH NEAR questionnaire [3]. Near and intermediate activity refers to the distance an object is from the eyes and may include paper reading, device use, painting, writing, or playing board games. The mean of the questionnaire-captured recall of near and intermediate activities was reported to be 10.34 ± 0.85 h/day but only 6.25 ± 0.39 h/day when captured from objective infrared glasses [3]. While there are limitations to the sensitivity of the objective measure, such as reduced accuracy at distances over 1 m, it highlights the likely impact of memory recall bias, in particular, overreporting. The presence of memory recall bias is also supported by the minimal utilisation of devices’ own data capture system reports by participants included in the Bland–Altman plot analysis between the EDUQ and 24DUD. Of the combined Australian and UK cohort EDUQ3 data, 68% of participants provided outcomes of device system reported screentime (predominantly smartphone reports), but only three participants indicated using these device reports to inform their answers to questions related to usual daily hours of device use. This suggests participants predominantly relied on memory to estimate daily hours of device use. The utilisation of the device reports did not appear to improve the agreement between the EDUQ and 24DUD, with similar differences occurring for these three participants than for all others. Whilst memory recall bias was hypothesised to be likely associated with the EDUQ during development, the magnitude of impact appeared far greater than anticipated. To evaluate memory recall bias, comparison of the EDUQ against a method such as direct observation may be required.

The poor agreement between the EDUQ and diaries may also indicate that eight 24DUDs are not adequate to capture ‘usual’ device use. With dietary intake 24-h recalls, it is known that increasing the number of recalls enables better capture of fluctuations in dietary intake, and thus, outcomes are more likely to be reflective of habitual intake [31]. Daily device use has high potential for day-to-day variability, as demonstrated by participants in this study. For example, one participant with a mean daily use of 6.6 h from eight 24DUDs reported only 0.5 h in one 24DUD (handheld device use) and 11.7 h in another 24DUD (5.58 h television, 4.00 h computer, 2.12 h handheld). It may be that a higher number of 24DUDs are needed to be representative of usual device use. Future studies may consider more days of diary capture or adapting dietary intake methods for device use such as the prospective dietary intake method of a three- or seven-day food record, or a diet history which includes in-depth retrospective capture by interview. In-depth interviewing or continuous capture may help to understand how device use varies between consecutive days. Additionally, future studies could look to investigate opportunities for using reports from the devices’ own data capture systems to support monitoring of behaviours across all device types and days of the week. In the present study smartphone and tablet reports were most utilised by participants. With any method selection, participant access to device reports, burden, and reactivity bias with a greater recording period are important considerations [32]. Future research may benefit from providing training or support to participants in how to efficiently record device use. Continued research to improve the validity of the EDUQ, or a similar questionnaire, would be beneficial, as it has the potential to be applied in multiple research areas. As mentioned earlier, it is of particular interest to understand any impacts of blue light exposure on macular health [5]. The EDUQ could also have applications in other areas of research interested in how device use may relate to population behaviours such as sleep and physical activity or psychological areas such as depression and body dissatisfaction [33, 34].

Multiple reasons may have contributed to the poor agreement between the EDUQ and 24DUD in this study. As a novel field of research, future studies looking to advance the validity and reliability of measurement of electronic device use behaviours may consider developing new instruments through grounded theory methodology [35, 36]. As seen in the present study, daily device use behaviours appear to be highly variable within and between individuals. Engaging with relevant population groups via focus groups and interviews to understand behaviours around electronic device use will likely be useful to inform the development of methods able to accurately capture electronic device use behaviours.

A number of limitations were present in this study. Convenience sampling resulted in a population that was predominantly young, highly educated, and female rather than representative of the general population. The UK cohort was smaller than the goal sample size, and the questionnaire incompletion rate was high. This was a limitation as it limited the ability to determine EDUQ validity through Bland–Altman plot agreement [27]. Future studies should look to increase the sample size and improve participant questionnaire completion rates, for example by reducing the participant burden with high questionnaire frequency. Another limitation was the use of relative validity with two unvalidated questionnaires as the method. Although access to an objective measure was not available, future studies may benefit from validating the 24DUD through comparison with direct behaviour observation or emerging objective technologies such as previously mentioned infrared glasses, known as the Clouclip and RangeLife glasses [3, 37]. Direct behaviour observation was not available as a comparative method in this study due to study design and data collection being conducted during the COVID-19 pandemic.

## Conclusions

This study reports on a novel tool developed specifically to monitor habitual patterns of electronic device use. The EDUQ demonstrated poor validity with poor agreement and ability to rank participants compared with mean daily hours of device use from multiple 24DUDs. Despite poor agreement, mean daily device use between each EDUQ and the 24DUDs were moderately to strongly correlated. This cohort was unable to consistently report similar device use between the third EDUQ and diaries, with misestimation appearing to occur across all device categories. To improve the validity of device use capture, future studies may benefit from a larger, more diverse sample size, the same reporting intervals for the tools being compared, and consideration of the time of year for data collection, as well as how an objective or direct observation method could be incorporated into the study design.

### Supplementary Information

**Additional file 1. **Describes the 22-item Electronic Device Use Questionnaire developed as part of this project.

**Additional file 2. **Describes the 24-hour electronic device use diary developed as part of this project.

**Additional file 3. **Described each of the calculations utilised for determining the mean daily hours of electronic device use for the Electronic Device Use Questionnaire and 24-hour electronic device use diary.

**Additional file 4. **Describes the outcomes of change in device use over last one to 20 years as reported in the Electronic Device Use Questionnaire completed in week one.

## Data Availability

The dataset supporting the conclusions of this article is available in the UQeSpace repository, 10.48610/61b97b1.

## Abbreviations

EDUQ: Electronic Device Use Questionnaire
UH NEAR: University of Houston Near work Environment Activity, and Refraction
UK: United Kingdom
24DUD: 24-hour electronic device use diary
