# Supplementary material for: Assessing electronic device use behaviours in healthy adults: development and evaluation of a novel tool
Source: BMC Public Health. 2024 Jan 15;24:186. doi: 10.1186/s12889-024-17637-4 (PMC10790453; doi:10.1186/s12889-024-17637-4)
Supplement: Supplementary file 1 — Additional file 1. Describes the 22-item Electronic Device Use Questionnaire developed as part of this project. [file 12889_2024_17637_MOESM1_ESM.docx]

Additional File 1

This additional file 1 describes the 22-item Electronic Device Use Questionnaire developed as part of this project.

**Electronic Device Use Questionnaire (EDUQ):**

The following questionnaire covers questions regarding general health, work history, education and physical activity behaviours. It also explores behaviours and habits surrounding electronic device use

Date: Name:

DOB:

Sex: Female Male Other, please specify:

Country of Residence: Post-code of Residence:

1. **Medical History:**

**1.1** Weight (kg):

**1.2** Height (cm):

**1.3** Do you have any chronic health conditions? (e.g. High blood pressure) Yes No

If yes please specify:

**1.4** Do you have any family history of a condition known as age-related macular degeneration? Yes No

If yes, please specify their relationship to you (e.g. mother):

**1.5** Do you have any family history of glaucoma? Yes No

If yes, please specify their relationship to you (e.g. mother):

**1.6** Do you have any family history of retinitis pigmentosa? Yes No

If yes, please specify their relationship to you (e.g. mother):

**1.7** Have you ever taken or are you currently taking a supplement that contains Lutein and/or Yes No

Zeaxanthin and/or Meso-zeaxanthin?

*Examples of common lutein/zeaxanthin/mesozeaxanthin supplements are*: Blackmores Lutein Defence, Blackmores Lutein Vision-Advanced, Blackmores MacuVision, Healthy Care Bilberry and Lutein, Wagner Bilberry and Lutein, Australian Natural Care Healthy Eyes, Ocuvite Lutein. *Note:*Multi-vitamins do not usually contain lutein/zeaxanthin/mesozeaxanthin. However, Swisse Ultivite contains lutein, if you take this please indicate this.

If yes, please specify the name of the supplement, when you have been taking the supplement and for how long:

**1.8** Please list any other medications or supplements you are currently taking:

_________________________________________________________________________________________________________________________________________________________________________________________________________________________________

1. **Education and Occupation:**

**2.1.** What highest level of education have you **completed**? (please tick)

___ Grade 10 School Completion

___ Grade 12 School Completion

___ TAFE certificate

___ TAFE diploma

**___** Trade apprenticeship certificate (e.g. carpentry)

___ Undergraduate University Degree

___ Masters University Degree

___ PhD

- 1. What is your **current** occupational status? (please tick)

___ Student

___ Employed / Self-Employed

___ On Leave (e.g. Maternity)

___ Unemployed

___ Retired

**2.3** Work history:

Please fill out the table below to provide information about your current and past professional occupations, (inclusive of casual/part-time/permanent). Only fill as many as needed or up to 20 years ago.

| Job Title (e.g. receptionist, coach, plumber, psychologist, sales assistant) | Type of work (e.g. admin, labourer, health, marketing, politics) | Number of months / years in role | I work outdoors over 50% of time in this role (Yes/No) | I look at electronic device screens over 50% of time in this role (Yes/No) |
| --- | --- | --- | --- | --- |
|  |  |  |  |  |
|  |  |  |  |  |
|  |  |  |  |  |
|  |  |  |  |  |
|  |  |  |  |  |
|  |  |  |  |  |
|  |  |  |  |  |
|  |  |  |  |  |

1. **Electronic Device Use:**

The following questions are about how you use electronic devices each day and how this has changed over your lifetime. For the purpose of this survey electronic devices include the following:

- Smartphones e.g. iPhone, Samsung, Huawei.
- Computers/laptops e.g. Dell, Microsoft, MacBook.
- Tablets e.g. Surface Pro, iPad.
- Television/Projector Screen e.g. TV, movie theatres, lecture/conference halls screens, meeting room screens.

***Note:*** For the purpose of this survey, using and electronic device is when you are looking at it and using it. For example, having the TV on in the background, but you are not actually looking directly at it does not count toward time using electronic devices.

**3.1** Thinking back over the last 3 months, please indicate the number of hours you normally spend performing the following activities on an average day.

**Weekday (i.e. Monday-Friday):**

Viewing a TV screen (e.g. movies, video games, news)

0 hrs

1 hr

2 hrs

3 hrs

4 hrs

5 hrs

6 hrs

7 hrs

8 hrs

9 hrs

10 hrs or more

Viewing a computer screen (e.g. laptop, desktop, computer games)

0 hrs

1 hr

2 hrs

3 hrs

4 hrs

5 hrs

6 hrs

7 hrs

8 hrs

9 hrs

10 hrs or more

Viewing a handheld electronic device (e.g. smartphone, tablet)

0 hrs

1 hr

2 hrs

3 hrs

4 hrs

5 hrs

6 hrs

7 hrs

8 hrs

9 hrs

10 hrs or more

**Weekend Day (i.e. Saturday and Sunday):**

0 hrs

1 hr

2 hrs

3 hrs

4 hrs

5 hrs

6 hrs

7 hrs

8 hrs

9 hrs

10 hrs or more

Viewing a TV screen (e.g. movies, video games, news)

Viewing a computer screen (e.g. laptop, desktop, computer games)

0 hrs

1 hr

2 hrs

3 hrs

4 hrs

5 hrs

6 hrs

7 hrs

8 hrs

9 hrs

10 hrs or more

0 hrs

1 hr

2 hrs

3 hrs

4 hrs

5 hrs

6 hrs

7 hrs

8 hrs

9 hrs

10 hrs or more

Viewing a handheld electronic device (e.g. smartphone, tablet)

*Adapted from Williams et al. (2019)*

**3.2** For each time point below please circle whether your daily use of electronic devices has either increased, decreased or remained the same:

| *Compared to* ***1*** *year ago my electronic device use has….*  Increased Decreased Not Changed | If it has **increased or decreased** please specify the main reason you think this may be: |
| --- | --- |
| *Compared to* ***5*** *years ago my electronic device use has…*    Increased Decreased Not Changed | If it has **increased or decreased** please specify the main reason you think this may be: |
| *Compared to* ***10*** *years ago my electronic device use has…*  Increased Decreased Not Changed | If it has **increased or decreased** please specify the main reason you think this may be: |
| *Compared to* ***15*** *years ago my electronic device use has…*  Increased Decreased Not Changed | If it has **increased or decreased** please specify the main reason you think this may be: |
| *Compared to* ***20*** *years ago my electronic device use has…*  Increased Decreased Not Changed | If it has **increased or decreased** please specify the main reason you think this may be: |

If your daily use of electronic device has **not changed** at all over the last 20 years, why do you think this may be?

**3.3** 24 Hour Recall

Thinking back to yesterday, please indicate how many hours you used the following electronic devices for the time brackets below (Morning, Afternoon, Evening):

**Morning (Waking to Midday)**

0 hrs

1 hr

2 hrs

3 hrs

4 hrs

5 hrs

6 hrs

7 hrs

8 hrs or more

Viewing a TV screen (e.g. movies, video games, news)

Viewing a computer screen (e.g. laptop, desktop, computer games)

0 hrs

1 hr

2 hrs

3 hrs

4 hrs

5 hrs

6 hrs

7 hrs

8 hrs or more

0 hrs

1 hr

2 hrs

3 hrs

4 hrs

5 hrs

6 hrs

7 hrs

8 hrs or more

Viewing a handheld electronic device (e.g. smartphone, tablet)

**Afternoon (Midday to 5pm)**

Viewing a TV screen (e.g. movies, video games, news)

0 hrs

1 hr

2 hrs

3 hrs

4 hrs

5 hrs

*Question continues over page…*

Viewing a computer screen (e.g. laptop, desktop, computer games)

0 hrs

1 hr

2 hrs

3 hrs

4 hrs

5 hrs

Viewing a handheld electronic device (e.g. smartphone, tablet)

0 hrs

1 hr

2 hrs

3 hrs

4 hrs

5 hrs

**Evening (5pm to Sleep)**

0 hrs

1 hr

2 hrs

3 hrs

4 hrs

5 hrs

6 hrs

7 hrs

8 hrs or more

Viewing a TV screen (e.g. movies, video games, news)

0 hrs

1 hr

2 hrs

3 hrs

4 hrs

5 hrs

6 hrs

7 hrs

8 hrs or more

Viewing a computer screen (e.g. laptop, desktop, computer games)

0 hrs

1 hr

2 hrs

3 hrs

4 hrs

5 hrs

6 hrs

7 hrs

8 hrs or more

Viewing a handheld electronic device (e.g. smartphone, tablet)

**3.4.1** Device reported screen time: If available, and turned on for your devices please indicate the screen time reports recorded by your device.

*(Apple devices found in ‘Settings’ under ‘Screen Time’, Google devices found under ‘Digital Wellbeing’, other Android devices may require you to have downloaded an application that tracks screen time)*

Please include information such as daily average, number of times you picked up or opened the device, and average time spent using different types of applications e.g. games, social networking, other.

**3.4.2** Did you use your devices screen time application to answer the previous questions? Yes No

If yes, please specify which questions below:

**3.5** Settings of electronic devices you use.

**3.5.1** Do you utilise settings on your electronic devices that change the colour of the screen to be more yellow?

On computers/laptops, tablets and smartphones there is often a setting available to change the colour of the screen to be more yellow (reduce blue light). It is usually aligned with your local sunset (turns more yellow) and sunrise (turns more blue).

Yes No

If yes, please specify below on what devices and when you use this setting.

**3.6.1** Do you wear glasses (for visual correction, reading etc.)? Yes No

*Note:* Even if you only wear glasses for certain activities please circle yes.

**3.6.2** If yes, when using electronic devices please circle how much of the time do you wear your glasses?

Never

Rarely

Some of the time

Most of the time

Always

**3.6.3** Does the lens of your glasses filter a portion of blue light entering your eye? Yes No Unsure

If yes, please specify below how much if known (e.g. filters 25% OR provide brand name of technology e.g. Baxter Blue).

1. **Physical Activity and Sleep**

***Note:*** For the purpose of this survey physical activity includes things such as walking, swimming, gardening, hiking, organised sport (e.g. netball, soccer) etc.

**4.1** How many times per week do you participate in leisure-time physical activity? ______

**4.2** How many hours per week do you participate in leisure-time physical activity? ______

**4.3** When outdoors do you were sunglasses… (please circle)

Never

Rarely

Some of the time

Most of the time

Always

**4.4** How many hours of sleep do you normally get on a week night? ______

Weekend night? ______

Thank you for completing this survey!

References:

1. Williams R, Bakshi S, Ostrin EJ, Ostrin LA. Continuous Objective Assessment of Near Work. Scientific reports. 2019;9(1):6901.
