# Supplementary material for: Assessing electronic device use behaviours in healthy adults: development and evaluation of a novel tool
Source: BMC Public Health. 2024 Jan 15;24:186. doi: 10.1186/s12889-024-17637-4 (PMC10790453; doi:10.1186/s12889-024-17637-4)
Supplement: Supplementary file 2 — Additional file 2. Describes the 24-hour electronic device use diary developed as part of this project. [file 12889_2024_17637_MOESM2_ESM.docx]

Additional File 2:

This additional file 2 describes the 24-hour electronic device use diary developed as part of this project.

**24-hour Electronic Device Use Diary**

| Time | | | Minutes Electronic Device Used | | |
| --- | --- | --- | --- | --- | --- |
|  |  |  | Viewing a TV screen (e.g. movies, video games, news) | Viewing a computer screen (e.g. laptop, desktop, computer games) | Viewing a handheld electronic device (e.g. smartphone, tablet) |
| 12:00:00 AM | to | 12:14:00 AM |  |  |  |
| 12:15:00 AM | to | 12:29:00 AM |  |  |  |
| 12:45:00 AM | to | 12:59:00 AM |  |  |  |
| 1:15:00 AM | to | 1:29:00 AM |  |  |  |
| 1:45:00 AM | to | 1:59:00 AM |  |  |  |
| 2:15:00 AM | to | 2:29:00 AM |  |  |  |
| 2:45:00 AM | to | 2:59:00 AM |  |  |  |
| 3:15:00 AM | to | 3:29:00 AM |  |  |  |
| 3:45:00 AM | to | 3:59:00 AM |  |  |  |
| 4:15:00 AM | to | 4:29:00 AM |  |  |  |
| 4:45:00 AM | to | 4:59:00 AM |  |  |  |
| 5:15:00 AM | to | 5:29:00 AM |  |  |  |
| 5:45:00 AM | to | 5:59:00 AM |  |  |  |
| 6:15:00 AM | to | 6:29:00 AM |  |  |  |
| 6:45:00 AM | to | 6:59:00 AM |  |  |  |
| 7:15:00 AM | to | 7:29:00 AM |  |  |  |
| 7:45:00 AM | to | 7:59:00 AM |  |  |  |
| 8:15:00 AM | to | 8:29:00 AM |  |  |  |
| 8:45:00 AM | to | 8:59:00 AM |  |  |  |
| 9:15:00 AM | to | 9:29:00 AM |  |  |  |
| 9:45:00 AM | to | 9:59:00 AM |  |  |  |
| 10:15:00 AM | to | 10:29:00 AM |  |  |  |
| 10:45:00 AM | to | 10:59:00 AM |  |  |  |
| 11:15:00 AM | to | 11:29:00 AM |  |  |  |
| 11:45:00 AM | to | 11:59:00 AM |  |  |  |
| 12:15:00 PM | to | 12:29:00 PM |  |  |  |
| 12:45:00 PM | to | 12:59:00 PM |  |  |  |
| 1:15:00 PM | to | 1:29:00 PM |  |  |  |
| 1:45:00 PM | to | 1:59:00 PM |  |  |  |
| 2:15:00 PM | to | 2:29:00 PM |  |  |  |
| 2:45:00 PM | to | 2:59:00 PM |  |  |  |
| 3:15:00 PM | to | 3:29:00 PM |  |  |  |
| 3:45:00 PM | to | 3:59:00 PM |  |  |  |
| 4:15:00 PM | to | 4:29:00 PM |  |  |  |
| 4:45:00 PM | to | 4:59:00 PM |  |  |  |
| 5:15:00 PM | to | 5:29:00 PM |  |  |  |
| 5:45:00 PM | to | 5:59:00 PM |  |  |  |
| 6:15:00 PM | to | 6:29:00 PM |  |  |  |
| 6:45:00 PM | to | 6:59:00 PM |  |  |  |
| 7:15:00 PM | to | 7:29:00 PM |  |  |  |
| 7:45:00 PM | to | 7:59:00 PM |  |  |  |
| 8:15:00 PM | to | 8:29:00 PM |  |  |  |
| 8:45:00 PM | to | 8:59:00 PM |  |  |  |
| 9:15:00 PM | to | 9:29:00 PM |  |  |  |
| 9:45:00 PM | to | 9:59:00 PM |  |  |  |
| 10:15:00 PM | to | 10:29:00 PM |  |  |  |
| 10:45:00 PM | to | 10:59:00 PM |  |  |  |
| 11:15:00 PM | to | 11:29:00 PM |  |  |  |
| 11:45:00 PM | to | 11:59:00 PM |  |  |  |

Adapted from physical activity diary by Cartmel et al. (1997)

References:

1. Cartmel B, Moon TE (1992) Comparison of two physical activity questionnaires, with a diary, for assessing physical activity in an elderly population. J Clin Epidemiol 45 (8):877-883. doi:10.1016/0895-4356(92)90071-t
