# Supplementary material for: Assessing electronic device use behaviours in healthy adults: development and evaluation of a novel tool
Source: BMC Public Health. 2024 Jan 15;24:186. doi: 10.1186/s12889-024-17637-4 (PMC10790453; doi:10.1186/s12889-024-17637-4)
Supplement: Supplementary file 3 — Additional file 3. Described each of the calculations utilised for determining the mean daily hours of electronic device use for the Electronic Device Use Questionnaire and 24-hour electronic device use diary. [file 12889_2024_17637_MOESM3_ESM.docx]

Additional File 3

Additional file 3 described each of the calculations utilised for determining the mean daily hours of electronic device use for the Electronic Device Use Questionnaire and 24-hour device use diary.

Table 1. Calculations for determining mean daily hours of electronic device use

| Tool | Electronic device category | Equation |
| --- | --- | --- |
| EDUQ | All devices | $\text{Mean daily hours of use from all devices combined = }$  $\text{(}\text{(weekday TV+ weekday computer + weekday handheld) × 5 + }$  $\text{(weekend TV + weekend computer + weekend handheld) × 2}\text{)}\text{ ÷7}$ |
|  | Television | $\text{Mean daily hours of use from TV= ((weekday TV × 5) +}$  $\text{(weekend TV × 2) ÷ 7}$ |
|  | Computer | $\text{Mean daily hours of use from computer = ((weekday computer }$  $\text{× 5) + (weekend computer × 2) ÷ 7}$ |
|  | Handheld | $\text{Mean daily hours of use from handheld = ((weekday handheld }$  $\text{× 5) +(weekend handheld × 2) ÷ 7}$ |
| 24-hour electronic device use diary | All devices | $\text{Mean daily hours of use from all devices combined = }$  $\text{(sum of use from all completed diaries for TV }$  $\text{+ computer + handheld) ÷ number of diaries completed }$ |
|  | Television | $\text{Mean daily hours of use from TV = (sum of use from all }$  $\text{completed diaries for TV) ÷ number of diaries completed}$ |
|  | Computer | $\text{Mean daily hours of use from computer = (sum of use from all }$  $\text{completed diaries for computer) ÷ number of diaries completed}$ |
|  | Handheld | $\text{Mean daily hours of use from handheld = (sum of use from all }$  $\text{completed diaries for handheld) ÷ number of diaries completed}$ |

Abbreviations: EDUQ, electronic device use questionnaire; TV, television
