# Supplementary material for: Assessing electronic device use behaviours in healthy adults: development and evaluation of a novel tool
Source: BMC Public Health. 2024 Jan 15;24:186. doi: 10.1186/s12889-024-17637-4 (PMC10790453; doi:10.1186/s12889-024-17637-4)
Supplement: Supplementary file 4 — Additional file 4. Describes the outcomes of change in device use over last one to 20 years as reported in the Electronic Device Use Questionnaire completed in week one. [file 12889_2024_17637_MOESM4_ESM.docx]

Additional File 4

Additional file 4 describes the outcomes of change in device use over last one to 20 years as reported in the Electronic Device Use Questionnaire completed in week one.

Table 2. Change in device use over last 1-20 years as per Electronic Device Use Questionnaire completed week one

| Aus n = 56  UK n = 24 | | 1 year ago | 5 years ago | 10 years ago | 15 years ago | 20 years ago |
| --- | --- | --- | --- | --- | --- | --- |
| Participants reporting increase (%) | Aus | 32 | 72 | 88 | 93 | 91 |
|  | UK | 50 | 88 | 92 | 96 | 100 |
| Participants reporting decrease (%) | Aus | 5 | 2 | 4 | 2 | 2 |
|  | UK | 8 | 0 | 0 | 0 | 0 |
| Participants reporting no change (%) | Aus | 63 | 26 | 9 | 5 | 7 |
|  | UK | 42 | 13 | 8 | 4 | 0 |

Abbreviations: Aus, Australia; UK, United Kingdom; n, number of participants.
